# Cancer Signaling Transcriptome Is Upregulated in Type 2 Diabetes Mellitus

**DOI:** 10.3390/jcm10010085

**Published:** 2020-12-29

**Authors:** Enrique Almanza-Aguilera, Álvaro Hernáez, Dolores Corella, Albert Sanllorente, Emilio Ros, Olga Portolés, Julieta Valussi, Ramon Estruch, Oscar Coltell, Isaac Subirana, Silvia Canudas, Cristina Razquin, Gemma Blanchart, Lara Nonell, Montserrat Fitó, Olga Castañer

**Affiliations:** 1 Cardiovascular Risk and Nutrition Research Group, Hospital del Mar Research Institute (IMIM), 08003 Barcelona, Spain; ealmanzaa@outlook.com (E.A.-A.); albertsanllorente@gmail.com (A.S.); jvalussimorresi@gmail.com (J.V.); gblanchart@imim.es (G.B.); mfito@imim.es (M.F.); 2 Centro de Investigación Biomédica en Red Fragilidad y Envejecimiento Saludable (CIBERFES), Instituto de Salud Carlos III, 28029 Madrid, Spain; 3 Institute of Nutrition and Food Safety (INSA-UB), University of Barcelona, 08921 Santa Coloma de Gramanet, Spain; 4 Cardiovascular Risk, Nutrition and Aging Research Unit, August Pi i Sunyer Biomedical Research Institute (IDIBAPS), 08036 Barcelona, Spain; alvaro.hernaez1@gmail.com (Á.H.); restruch@clinic.cat (R.E.); 5 Blanquerna School of Life Sciences, Universitat Ramón Llull, 08025 Barcelona, Spain; 6 Centro de Investigación Biomédica en Red Fisiopatologia de la Obesidad y la Nutrición (CIBEROBN), Instituto de Salud Carlos III, 28029 Madrid, Spain; dolores.corella@uv.es (D.C.); eros@clinic.cat (E.R.); olga.portoles@uv.es (O.P.); oscar.coltell@uji.es (O.C.); silvia.canudas@gmail.com (S.C.); crazquin@unav.es (C.R.); 7 Department of Preventive Medicine, University of Valencia, 46010 Valencia, Spain; 8 Department of Internal Medicine, Hospital Clínic, Institut d’Investigacions Biomèdiques August Pi Sunyer (IDIBAPS), University of Barcelona, 08036 Barcelona, Spain; 9 Department of Computer Languages and Systems, University Jaume I, 12071 Castellon, Spain; 10 Cardiovascular Epidemiology and Genetics Research Group, Hospital del Mar Research Institute (IMIM), 08003 Barcelona, Spain; isubirana@imim.es; 11 Centro de Investigación Biomédica en Red Epidemiología y Salud Pública (CIBERESP), Instituto de Salud Carlos III, 28009 Madrid, Spain; 12 Human Nutrition Department, Hospital Universitari Sant Joan, Institut d’Investigació Sanitària Pere Virgili, University Rovira i Virgili, 43204 Reus, Spain; 13 Department of Preventive Medicine and Public Health, IdiSNA, Navarra Institute for Health Research, University of Navarra, 31008 Pamplona, Spain; 14 Microarrays Analysis Service, Hospital del Mar Research Institute (IMIM), 08003 Barcelona, Spain; lnonell@imim.es

**Keywords:** transcriptome, microarray, signaling pathways, cancer, type 2 diabetes

## Abstract

We aimed to explore the differences in the whole transcriptome of peripheral blood mononuclear cells between elderly individuals with and without type 2 diabetes (T2D). We conducted a microarray-based transcriptome analysis of 19 individuals with T2D and 15 without. Differentially expressed genes according to linear models were submitted to the Ingenuity Pathway Analysis system to conduct a functional enrichment analysis. We established that diseases, biological functions, and canonical signaling pathways were significantly associated with T2D patients when their logarithms of Benjamini–Hochberg-adjusted *p*-value were >1.30 and their absolute z-scores were >2.0 (≥2.0 meant “upregulation” and ≤ −2.0 “downregulation”). Cancer signaling pathways were the most upregulated ones in T2D (z-score = 2.63, −log(*p*-value) = 32.3; 88.5% (*n* = 906) of the total differentially expressed genes located in these pathways). In particular, integrin (z-score = 2.52, −log(*p*-value) = 2.03) and paxillin (z-score = 2.33, −log(*p*-value) = 1.46) signaling pathways were predicted to be upregulated, whereas the Rho guanosine diphosphate (Rho-GDP) dissociation inhibitor signaling pathway was predicted to be downregulated in T2D individuals (z-score = −2.14, −log(*p*-value) = 2.41). Our results suggest that, at transcriptional expression level, elderly individuals with T2D present an increased activation of signaling pathways related to neoplastic processes, T-cell activation and migration, and inflammation.

## 1. Introduction

Type 2 diabetes (T2D) is a metabolic disorder characterized by chronic insulin resistance and subsequent hyperglycemia. It is a major risk factor for fatal and non-fatal cardiovascular disease [1], neurodegeneration [2], and cancer [3]. Several inter-related mechanisms, including oxidative stress, inflammation, and dysregulation of cell dynamics, have been proposed as the main links between T2D and chronic disease [4,5]. Establishing these mechanisms could contribute to better understanding of T2D evolution and the treatment of its complications. *Omics* technologies, including transcriptomics, proteomics, and metabolomics, have emerged as promising tools in this field [6]. Transcriptomics, in particular, help to identify molecular networks impaired in T2D [7]. Nevertheless, the relevant tissues are often unavailable and gene expression among tissues is relatively variable [8]. To overcome such limitations, the use of peripheral blood mononuclear cells (PBMC) has been extensively proposed in the literature [9]. They are circulating immune cells (T and B lymphocytes, natural killers, and monocytes) that together express up to 80% of the encoded human genome [10] and play a key role in the inflammatory processes involved in the physiopathology of T2D [11] and its complications [12]. In the current work, we hypothesized that the whole transcriptome of PBMC would not only reflect the pathophysiology of T2D, but also elucidate molecular dysregulations potentially related to the onset of T2D-related complications. 

The aim of the present work was to comprehensively analyze the transcriptome of PBMC from individuals with and without T2D, and subsequently conduct a functional enrichment analysis to determine the association between T2D and the activation of diseases, biological functions, and canonical signaling pathways.

## 2. Experimental Section

### 2.1. Study Design and Participants

**Figure 1:**
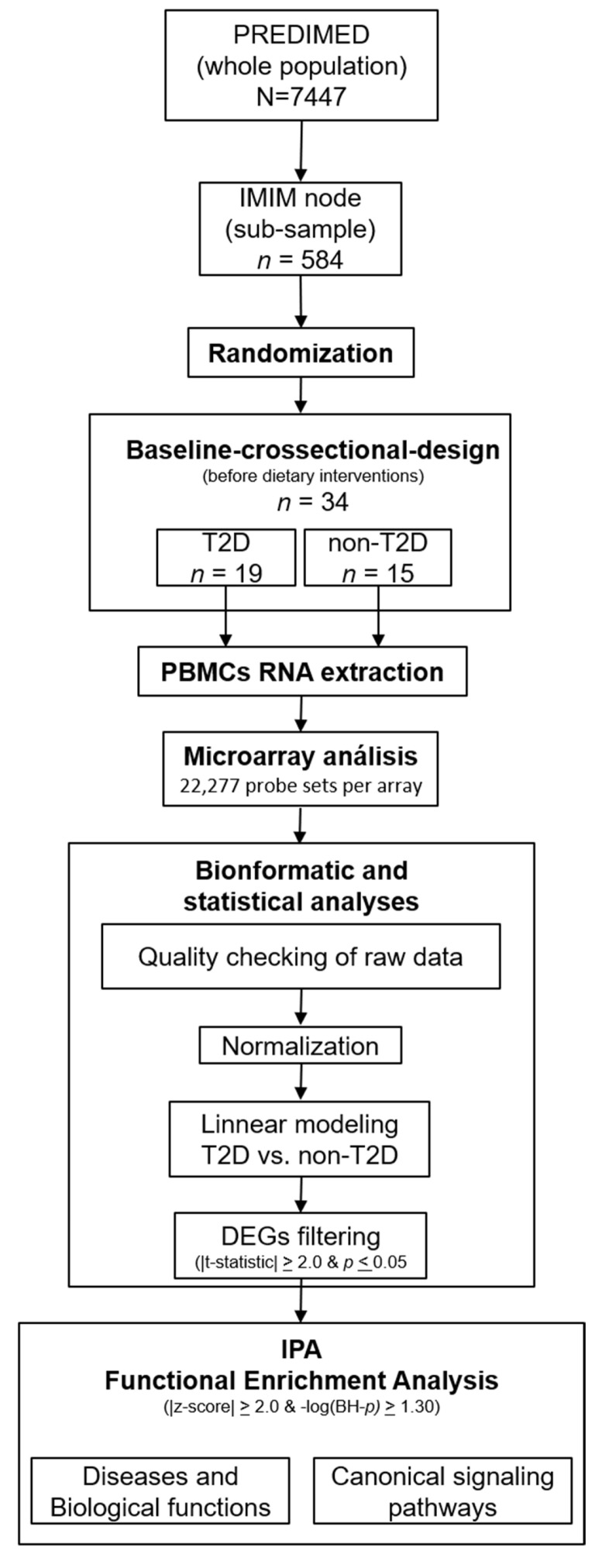
Participant and data analysis flowchart followed in the current study. Abbreviations: BH-P, Benjamini–Hochberg-adjusted *p* value; DEGs, differentially expressed genes; IMIM, Hospital del Mar Research Institute; IPA, Ingenuity Pathway Analysis; non-T2D, non-type 2 diabetes; PBMC, peripheral blood mononuclear cells; PREDIMED, Prevención con Dieta Mediterránea; T2D, type 2 diabetes.

This research followed a baseline, cross-sectional study design and included a subset of 34 participants from the PREDIMED (Prevención con Dieta Mediterránea) study, a clinical trial aimed at assessing the effectiveness of the traditional Mediterranean diet on the primary prevention of cardiovascular disease. Between June 2007 and October 2008, subjects of the current study (*n* = 34) were randomly selected from a total of 584 recruited at the Instituto Hospital del Mar de Investigaciones Médicas (Barcelona) PREDIMED center. They were community-dwelling men (55–80 years) and women (60–80 years) free from cardiovascular disease at enrollment but presenting either T2D or >3 of the following risk factors: current smoking, hypertension, high levels of low-density lipoprotein cholesterol, low concentrations of high-density lipoprotein cholesterol, overweight/obesity, or a family history of premature coronary heart disease. They were then grouped according to whether they presented T2D (*n* = 19) or not (non-T2D, *n* = 15) at baseline. T2D was defined by clinical diagnosis or use of antidiabetic medication [13,14]. At the time of recruitment participants with T2D received both or either antidiabetic medications (i.e., oral or insulin) and/or lifestyle-based management programs for blood glucose control. A flowchart showing the participants included in the current substudy, and the transcriptome functional algorithm analysis followed, is depicted in Figure 1. The study protocol complied with the Declaration of Helsinki, was approved by local institutional review boards, and was registered under the International Standard Randomized Controlled Trial Number ISRCTN35739639 (http://www.isrctn.com/ISRCTN35739639). All participants provided written informed consent before joining the trial. Full details of the study design, inclusion and exclusion criteria, and dietary intervention have been published elsewhere [14,15].

### 2.2. Biosample Collection and Measurements

Blood samples were collected after an overnight fast, aliquoted, coded, and either used for biochemical measurements within the first 12 h or frozen at −80 °C until microarray experiments. Biochemical determinations, including serum glucose and lipid profile, were performed by standard methods [16]. Trained personnel performed the anthropometric and blood pressure measurements. All participants were required to complete validated questionnaires to record disease history and medication use [15]. 

### 2.3. Microarray Experiments

Blood sample processing, RNA extraction, and microarray experiment procedures were performed as described by Castañer et al. [16]. In brief, mononuclear cells were isolated from peripheral blood with cell preparation tubes (Becton Dickinson, Franklin Lakes, NJ, USA) combining differential centrifugations and buffer washings, resuspended in Ultraspec RNA Isolation Reagent (Bioteck Laboratories, Hyderabad, Telangana), and finally stored at –80 °C until RNA isolation. Once isolated, RNA was assessed for concentration and purity with UV spectroscopy (A260) (NanoDrop ND-1000; NanoDrop Technologies), and for integrity using microcapillary gel electrophoresis (Bioanalyzer, NanoChip; Agilent Technologies). Gene expression profiling was performed with the customized GeneChip™ Human Genome U133A 2.0 array (Thermofishet Scientific), a commercial microarray platform that analyzes the expression level of 18,400 transcripts and variants, including 14,500 well-characterized human genes. Data from the current microarray were recorded in the *Gene Expression Omnibus* repository under the GSE28358 access register and were validated by quantitative real-time polymerase chain reaction, as reported by Castañer et al. [16]. All microarray experiments, and the subsequent transcriptome data processing and analyses, were performed at the Príncipe Felipe Research Centre, Valencia, Spain. 

### 2.4. Bioinformatic Analyses

We included all 22,277 probe sets represented in the array in the comparison analyses (Figure 1). The raw signal intensities from microarray experiments were background corrected, log_2_ transformed, and then quantile-normalized using Robust Multi-array Average methodology [17]. Subsequently, a principal component analysis and clustering plots were conducted to assess data distribution. In order to identify differentially expressed genes between T2D and non-T2D, linear models were conducted with the *Limma* R/Bioconductor software package [18]. In this way, a moderated t-statistic (*t*), *p* value, and log_2_-fold changes (log_2_-FC) were calculated for each gene. Genes were considered differently expressed when they presented absolute t-statistics ≥ 2.0 and *p* values ≤ 0.05. All the statistical procedures included in this stage were performed using the computing environment R version 2.15.1 (R Development Core Team, 2012).

### 2.5. Functional Enrichment Analysis

Functional enrichment analysis, including the prediction of diseases, biological functions, and signaling canonical pathways associated with T2D, was performed using Ingenuity Pathway Analysis software (IPA; Qiagen, Redwood City, CA, USA) [19]. An input file containing the differentially expressed genes, moderate t-statistics, and *P* and log_2_-FC values was loaded into the software. The core-expression analysis function, with the “Ingenuity Knowledge Base (Genes only)” as reference set, was then applied. The statistical significance and direction of activation for each disease, biological function, and signaling canonical pathway were determined using two metrics: the z-score and the adjusted *p* value. The z-score is a statistical measure that provides predictions about whether a biological process is upregulated (positive z-score) or downregulated (negative z-score). The *p* value, according to the right-tailed Fisher’s exact test, reflects the likelihood of the association between a set of genes and a determined biological process being significant. Furthermore, to control for a false discovery rate among biological process modulations, the software provides an adjustment of raw *p* values through the Benjamini–Hochberg (BH) procedure. In the current work, we considered that a disease, biological function, or canonical pathway was significantly associated with T2D when it showed both a z-score ≥ 2.0 and a BH-adjusted *p* value ≤ 0.05 (−log(BH-*P*) ≥ 1.30) [19].

### 2.6. Sample Size and Power Analysis

We estimated that a total sample of 34 participants allowed ≥80% of power to detect at least 5% of true differentially expressed genes between non-T2D and T2D conditions. Calculations were based on the algorithm proposed by Lin et al. [20], assuming a 95% probability of detecting such power and proportion of true differentially expressed genes, a false discovery rate level of 0.05, and log_2_-FC ≥ 2.0

### 2.7. Analysis of Anthropometric and Clinical Data

The Kolmogorov–Smirnov test and normal probability plots were used to determine the distribution of anthropometric and clinical data. Variables with a non-normal distribution were log-transformed prior to analysis. Differences in continuous and categorical variables between T2D and non-T2D groups were assessed by *t*-tests and chi-squared tests, respectively. Statistical analysis of anthropometric and clinical data was conducted with the SPSS 22.0 software (IBM SPSS Statistics for Windows. Armonk, NY, USA: IBM Corp.). A *p* ≤ 0.05 value was considered to be statistically significant in all tests.

## 3. Results

### 3.1. Participants’ Characteristics

The characteristics of the participants are presented in Table 1. As expected, T2D individuals presented higher serum glucose levels (+48 mg/dL) and greater use of oral antidiabetics and insulin. In addition, the T2D group had 45.9% less hypercholesterolemia and 21.4% fewer smokers in comparison with non-T2D.

### 3.2. Microarray Gene Expression and Functional Enrichment Analysis

**Figure 2:**
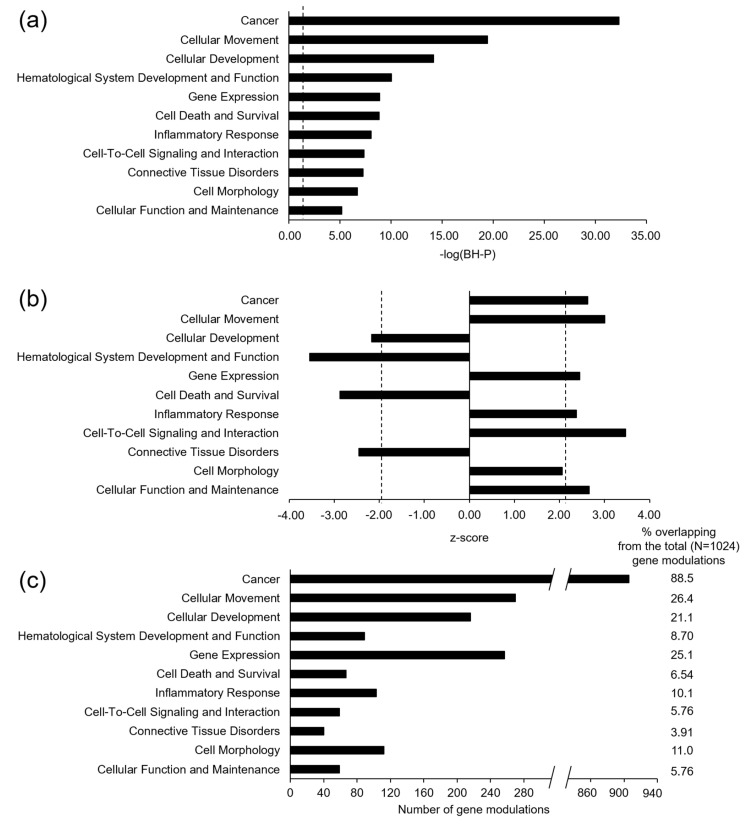
Diseases and biological functions predicted to be modulated in elderly individuals with T2D (**a**,**b**), and the number of genes involved in each of them (**c**). Significant modulations were defined by -logarithm of Benjamini–Hochberg-adjusted *p* value (−log(BH-*P*)) ≥ 1.30 (**a**) and an absolute z-score ≥ 2.0 (**b**) (vertical dashed lines, where appropriate).

We found 1024 differentially expressed genes between T2D and non-T2D individuals (569 were upregulated and 455 downregulated). Gene expression linked to 11 diseases and biological functions was predicted to be activated in T2D patients (Figure 2a,b). 

Specifically, cancer (z-score = 2.63, −log(BH-*P*) = 32.3), cellular movement (z-score = 3.01, −log(BH-*P*) = 19.4), gene expression (z-score = 2.45, −log(BH-*P*) = 8.88), inflammatory response (z-score = 2.37, −log(BH-*P*) = 8.03), cell-to-cell signaling and interaction (z-score = 3.47, −log(BH-*P*) = 7.36), cell morphology (z-score = 2.06, −log(BH-*P*) = 6.69), and cellular function and maintenance (z-score = 2.66, −log(BH-*P*) = 5.17) were predicted to be upregulated. In contrast, cellular development (z-score = −2.17, −log(BH-*P*) = 14.2), hematological system development and function (z-score = −3.55, −log(BH-*P*) = 10.0), cell death and survival (z-score = −2.87, −log(BH-*P*) = 8.81), and connective tissue disorders (z-score = −2.45, −log(BH-*P*) = 7.24) were predicted to be downregulated. According to the number of genes involved (*n* = 906) and the overlapping percentage (88.5%) of all differentially expressed genes, cancer signaling was the most activated in T2D patients (Figure 2c). 

**Figure 3:**
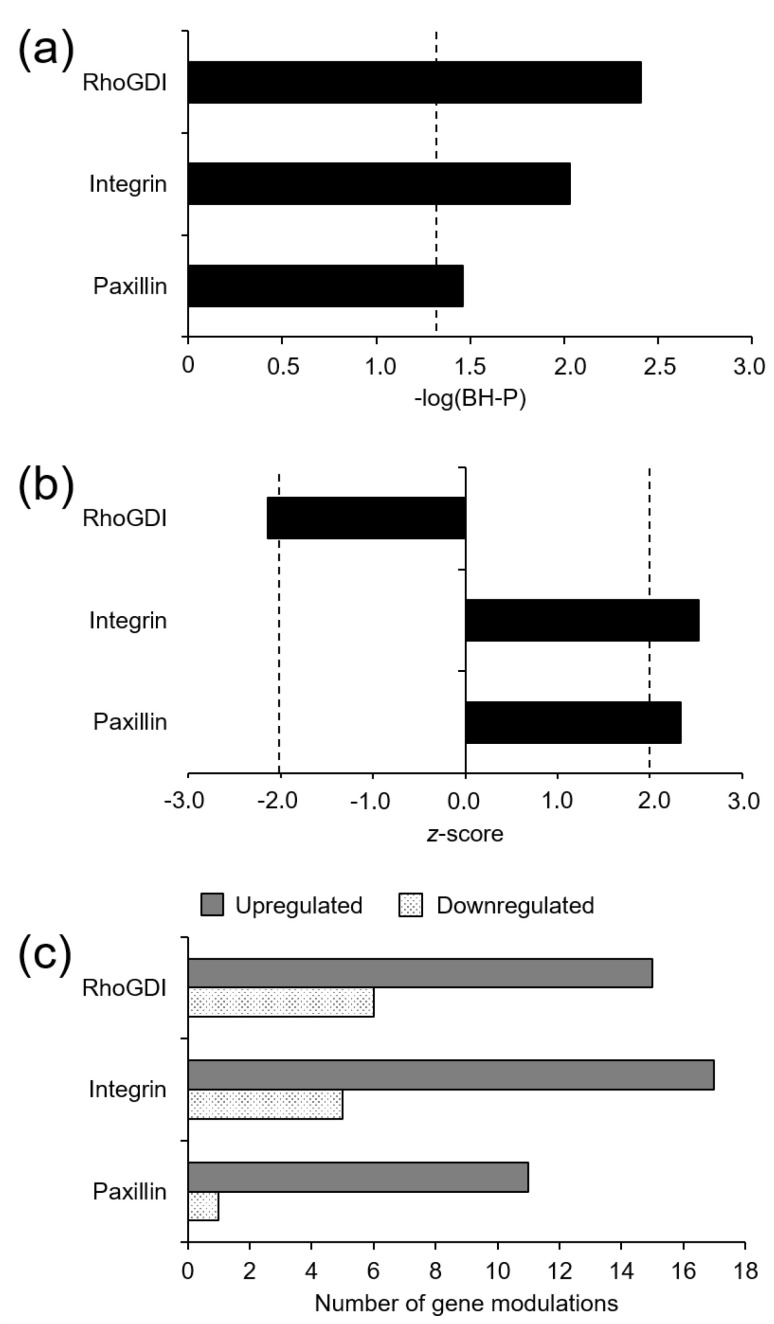
Canonical signaling pathways predicted to be modulated in elderly individuals with T2D (**a**,**b**), and number of genes modulated in each of them (**c**). Significant modulations were defined by −logarithm of Benjamini–Hochberg-adjusted *p* value (−log(BH-*P*)) ≥ 1.30 (**a**) and an absolute z-score ≥ 2.0 (**b**) (vertical dashed lines, where appropriate).

The Ingenuity Pathway Analysis (IPA) system also predicted the activation of three signaling canonical pathways in T2D. Integrin (z-score = 2.52, −log(BH-*P*) = 2.03; genes: 18 upregulated, 4 downregulated) and paxillin (z-score = 2.33, −log(BH-*P*) = 1.46; genes: 11 upregulated, 1 downregulated) signaling pathways were predicted to be upregulated, whereas the Rho guanosine diphosphate ssociation inhibitor (RhoGDI)signaling pathway was predicted to be downregulated in T2D (z-score = −2.14, −log(BH-*P*) = 2.41; genes: 16 upregulated, 5 downregulated) (Figure 3).

**Figure 4:**
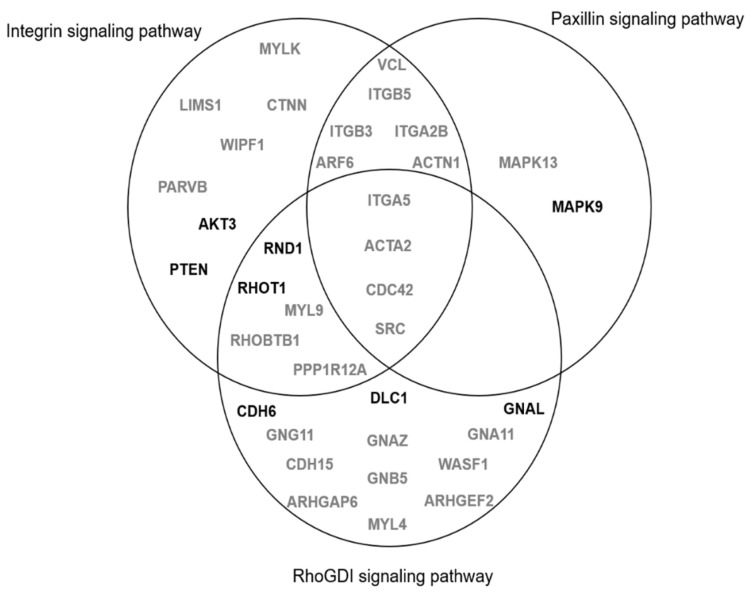
Exclusive and common genes downregulated (bold) and upregulated (gray) among integrin, paxillin, and Rho guanosine diphosphate dissociation inhibitor (RhoGDI) signaling pathways predicted to be modulated in elderly individuals with T2D. MYLK, myosin light chain kinase; ACTN1, actinin alpha 1; ACTA2, actin alpha 2; AKT3, AKT serine/threonine kinase 3; ARF6, ADP ribosylation factor 6; ARHGAP6, Rho GTPase activating protein 6; ARHGEF2, Rho/Rac guanine nucleotide exchange factor 2; CDC42, cell division cycle 42; CDH6, cad-herin 6; CDH15, cadherin 15; CTNN, beta catenin; DLC1, DLC1 Rho GTPase activating protein; GNA11, G protein subunit alpha 11; GNAZ, G protein subunit alpha z; GNAL, G protein subunit alpha L; GNB5, G protein subunit beta 5; GNG11, G protein subunit gamma 11; ITGB3, integrin subunit beta 3; ITGB5, integrin subunit alpha 5; ITGA2B, in-tegrin subunit alpha 2b; ITGB5, integrin subunit beta 5; LIMS1, LIM zinc finger domain containing 1; MAPK13, mitogen-activated protein kinase 9; MAPK13, mitogen-activated protein kinase 13; MYL4, myosin light chain 4; MYL9, myosin light chain 9; PTEN, phos-phatase and tensin homolog; WIPF1, WAS/WASL interacting protein family member 1; PARVB, parvin beta; PPP1R12A, protein phosphatase 1 regulatory subunit 12A; RND1, Rho family GTPase 1; RHOT1, ras homolog family member T1; RHOBTB1, Rho related BTB domain containing 1; SRC, SRC proto-oncogene, non-receptor tyrosine kinase; VCL, vinculin; WASF1, WASP family member 1.

Distribution of gene expression changes among these signaling pathways is shown in Figure 4. 

**Figure 5:**
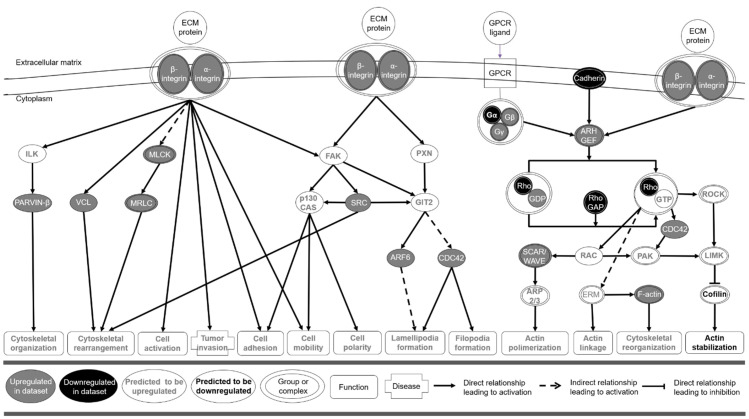
Predicted relationship between expressed and regulated genes within integrin, paxillin, and RhoGDI signaling pathways, and the consequent activation of diseases and biological functions in elderly individuals with T2D. The figure was adapted from the corresponding individual canonical signaling pathways generated by the Ingenuity Pathway Analysis system. ARF6, ADP-ribosylation factor 6; ARH, low density lipoprotein receptor adaptor protein 1; CDC42, Cell division control protein 42 homolog; ECM, extracellular matrix; ERM, ezrin/radixin/moesin; FAK, focal adhesion kinase; GEF, guanine nucleotide exchange factor; GIT2, G protein-coupled receptor kinase-interactor 2 isoform 4; GPCR, G protein-coupled receptors; GTP, guanosine triphosphate; ILK, Integrin-linked protein kinase; LIMK, lim kinase; MLCK, Myosin light-chain kinase; MRLC, myosin regulatory light chain; PAK; P21 activated kinase; PXN, paxillin; Rho GAP, Rho GTPase-activating proteins; VCL, vinculin.4. Discussion.

According to the IPA, the upregulation of integrin and paxillin signaling pathways, and the downregulation of RhoGDI signaling pathway, in T2D would lead to the activation of cytoskeletal organization, rearrangement and reorganization, cell activation, adhesion, mobility and polarity, lamellipodia and filopodia formation, and actin polymerization and linkage, and the inhibition of actin stabilization (Figure 5).

In the current study, we conducted a microarray-based transcriptome analysis in elderly individuals with and without T2D, and identified diseases, biological functions, and canonical pathways differentially expressed in T2D. We found that cancer signaling and biological functions, and canonical pathways related to cell dynamics, were upregulated in T2D patients.

T2D has been related with an increased risk of developing a wide set of malignancies [3,21,22]. In addition, a large amount of robust and unbiased epidemiological and clinical evidence suggests that it is associated with not only an increased risk for cancer incidence and mortality [3,23,24], but also several cancer types including breast, colorectum, intrahepatic cholangiocarcinoma, and endometrium. Such findings concur with our results, which indicate that an upregulation of cancer disease signaling is positively linked with T2D and that, compared with non-T2D, T2D individuals could have an increased risk of initiating neoplastic processes. 

Obesity and the use of glucose-lowering medications, two T2D-associated conditions, have been shown to be relevant modulators of the transcriptome in PBMC [25,26]. Obesity is strongly associated with greater esophageal, colorectal, liver, pancreatic, postmenopausal breast, endometrial, renal, gastric cardia, gallbladder, and ovary cancer risk [27,28]. Regarding antidiabetic medications, epidemiological studies suggest that whereas metformin could act as antineoplastic agent, therapies increasing circulating levels of insulin (i.e., sulfonylureas and exogenous insulin) could be related to higher cancer risk [29]. On the other side, regarding the signaling effects of antidiabetic medications, conflicting data have been published suggesting that, although their use could potentially enhance proliferation of premalignant or malignant cells, it is extremely unlikely they introduce malignancy [30]. All these conditions have been proposed as mediators of the relationship between T2D and cancer development [31], as well as other factors such as oxidative stress, chronic inflammation, and impaired levels of sex hormones [31,32,33]. 

Our results showing that PBMC transcriptome could be a suitable model to unravel the relationship between T2D and cancer are consistent with others previously reported in literature. In a recent study conducted by Calimlioglu and colleagues (2015), transcriptomics data from different tissues including beta-cells, pancreatic islets, arterial tissue, PBMC, liver, and skeletal muscle of 228 samples were integrated with protein–protein interaction data and genome-scale metabolic models to unravel the molecular and tissue-specific biomarker signatures of T2D [34]. Interestingly, authors found that with exception of beta cells and arterial tissues, cancer signaling pathways were upregulated in all tissues.

Integrin, paxillin, and RhoGDI signaling pathways are intimately inter-related and involved in cell dynamics [35]. Integrins, which mediate cell adhesion and the transduction of external signals to the actin cytoskeleton [36], have been shown to contribute to cancer progression and drive the therapeutic resistance of some malignancies [37,38]. Paxillin is a regulator of cytoskeleton structure involved in cell attachment, spreading, and migration [39], and is known to acquire gain-of-function mutations associated with the progression of many tumors such as colorectal and pancreatic ones [40]. Finally, RhoGDI dissociation inhibitor proteins modulate Rho-guanosine triphosphatases (involved in the regulation of cell adhesion, spreading, migration, polarity, survival, and division) [35]. The underexpression of these proteins promotes cell migration [41] and has been found in colorectal [42] and pancreatic cancers [43]. According to our findings, the upregulation of integrin and paxillin, and the downregulation of RhoGDP dissociation inhibitor signaling pathways, in individuals with T2D could be associated with the promotion of biological processes involved in cell activation and migration (e.g., cell movement and morphology and cell-to-cell signaling and interaction) and greater inflammatory responses. Our results agree with previous evidence indicating that diabetes and chronic hyperglycemia are linked to the upregulation of integrin and paxillin pathways and the downregulation of RhoGDP dissociation inhibitor responses [44,45,46] and the exacerbated T-cell activation and chronic low-grade inflammation present in T2D and its complications [47,48]. 

Our study has some limitations. First, its design was cross-sectional, which only allowed associations between T2D and differences in transcriptome to be established. Our conclusions and their clinical implications regarding cancer should, therefore, be corroborated in further studies in humans. Second, it had a relatively small sample size, which may have limited our statistical power. In order to counteract this limitation and add robustness to our results, we employed systematic bioinformatic procedures and functional enrichment analyses, in addition to adjusting for multiple comparisons. Finally, the presence of other cardiovascular risk factors beyond T2D complicates the extrapolation of our conclusions to other populations. 

## 4. Conclusions

In summary, T2D was associated with the upregulation of pathways related to cancer signaling, T-cell activation and migration, and inflammation. Our findings provide, from a transcriptomic point of view, insights concerning T2D pathophysiology and complications in a group of elderly adults at high cardiovascular risk. The modulation of present signaling pathways would be pharmacological targets in the primary prevention of development of neoplastic processes under T2D condition.

## Figures and Tables

**Table 1:** Characteristics of study participants.

	T2D (*n* = 19)	Non-T2D (*n* = 15)	*p*
Age, years	65 ± 6	63 ± 4	0.37
Female, *n* (%)	11 (57.9)	8 (53.3)	0.79
Weight, kg	81.0 ± 10.2	75.2 ± 14.3	0.18
BMI, kg/m^2^	30.1 ± 3.10	28.5 ± 4.35	0.22
Serum glucose, mg/dL	151 ± 55.2	103 ± 20.6	<0.01
Tobacco use, *n* (%)			0.02
Regular smoker	1 (5.30)	4 (26.7)	
Former smoker (>1y)	4 (21.1)	7 (46.7)	
Never smoker	14 (73.7)	4 (26.7)	
Hypertension, *n* (%)	13 (68.4)	12 (80.0)	0.70
Hypercholesterolemia, *n* (%)	9 (47.4)	14 (93.3)	0.01
Medication use, *n* (%)			
Cardiovascular drugs	2 (11.1)	1 (7.10)	0.70
Antihypertensive	13 (68.4)	9 (60.0)	0.72
Hypocholesterolemic	6 (31.6)	5 (33.3)	0.91
Oral antidiabetics	13 (68.4)	0.00	<0.01
Insulin	1 (5.26)	0.00	<0.01

Data are shown as mean ± standard deviation or *n* (%), as appropriate. Differences between groups were assessed by *t*-tests and chi-square test for continuous and categoric variables, respectively.

## Data Availability

Please contact corresponding author for data requests.
